# Blood Culture Testing via a Mobile App That Uses a Mobile Phone Camera: A Feasibility Study

**DOI:** 10.2196/jmir.6398

**Published:** 2016-10-26

**Authors:** Guna Lee, Yura Lee, Yong Pil Chong, Seongsoo Jang, Mi Na Kim, Jeong Hoon Kim, Woo Sung Kim, Jae-Ho Lee

**Affiliations:** ^1^Medical Information OfficeAsan Medical CenterUniversity of Ulsan College of MedicineSeoulRepublic of Korea; ^2^Department of Biomedical InformaticsAsan Medical CenterUniversity of Ulsan College of MedicineSeoulRepublic of Korea; ^3^Department of Infectious DiseasesAsan Medical CenterUniversity of Ulsan College of MedicineSeoulRepublic of Korea; ^4^Department of Laboratory MedicineAsan Medical CenterUniversity of Ulsan College of MedicineSeoulRepublic of Korea; ^5^Department of Pulmonary & Critical Care MedicineAsan Medical CenterUniversity of Ulsan College of MedicineSeoulRepublic of Korea; ^6^Department of Emergency MedicineAsan Medical CenterUniversity of Ulsan College of MedicineSeoulRepublic of Korea

**Keywords:** blood specimen collection, patient safety, mobile applications, mobile phone, user-computer interface, bar codes, patient identification systems

## Abstract

**Background:**

To evaluate patients with fever of unknown origin or those with suspected bacteremia, the precision of blood culture tests is critical. An inappropriate step in the test process or error in a parameter could lead to a false-positive result, which could then affect the direction of treatment in critical conditions. Mobile health apps can be used to resolve problems with blood culture tests, and such apps can hence ensure that point-of-care guidelines are followed and processes are monitored for blood culture tests.

**Objective:**

In this pilot project, we aimed to investigate the feasibility of using a mobile blood culture app to manage blood culture test quality. We implemented the app at a university hospital in South Korea to assess the potential for its utilization in a clinical environment by reviewing the usage data among a small group of users and by assessing their feedback and the data related to blood culture sampling.

**Methods:**

We used an iOS-based blood culture app that uses an embedded camera to scan the patient identification and sample number bar codes. A total of 4 medical interns working at 2 medical intensive care units (MICUs) participated in this project, which spanned 3 weeks. App usage and blood culture sampling parameters (including sampler, sampling site, sampling time, and sample volume) were analyzed. The compliance of sampling parameter entry was also measured. In addition, the participants’ opinions regarding patient safety, timeliness, efficiency, and usability were recorded.

**Results:**

In total, 356/644 (55.3%) of all blood culture samples obtained at the MICUs were examined using the app, including 254/356 (71.3%) with blood collection volumes of 5-7 mL and 256/356 (71.9%) with blood collection from the peripheral veins. The sampling volume differed among the participants. Sampling parameters were completely entered in 354/356 cases (99.4%). All the participants agreed that the app ensured good patient safety, disagreed on its timeliness, and did not believe that it was efficient. Although the bar code scanning speed was acceptable, the Wi-Fi environment required improvement. Moreover, the participants requested feedback regarding their sampling quality.

**Conclusions:**

Although this app could be used in the clinical setting, improvements in the app functions, environment network, and internal policy of blood culture testing are needed to ensure hospital-wide use.

## Introduction

Owing to the increase in the widespread use of mobile phones and improvements in wireless networks, the role of mobile health (mHealth) is growing [1-3]. By using this service, patients can be cared for by health care providers at any location and at any time, thus overcoming the limitations of time and space [4,5]. mHealth can help to realize the advantages of health information technology in point-of-care settings [6-8]. In particular, this service can provide information on drugs and diseases and can support clinical decisions [9-11]. Moreover, mHealth—a useful tool for both patients and health care providers—can serve as a tool to overcome the limitations of conventional medical services [3,5]. The services available via mHealth include monitoring of an individual’s condition, collection of health data, and prediction of health problems [2,3,5,6,8]. mHealth can also affect the decisions of physicians based on certain algorithms and can provide them with patient data. Thus, this service enables clinicians to make rapid and precise decisions by reducing errors and facilitates convenience in data access [1,8]. Furthermore, mHealth can be used for quality improvement at tertiary hospitals, wherein considerable information and recommendations are exchanged between patients and clinicians [1,8,12].

To evaluate patients with fever of unknown origin or those with suspected bacteremia, the precision of blood culture tests is critical [13-16]. The processes and parameters for blood culturing should strictly adhere to the guidelines of blood culture tests [14,17-20]. Among the parameters for blood cultures, sample volume [14,18], sampling site [12,21], and sampling time [22] are the most important factors affecting the sensitivity and specificity for detecting organisms in the bloodstream. An inappropriate step in the test process or error in a parameter could also lead to a false-positive result, which could then affect the direction of treatment in critical conditions [14,23-27]. As many clinicians are unaware of these guidelines, it is important to monitor the test process for better management and improved quality [28]. mHealth apps can be used to resolve problems with blood culture tests [29], and such apps can hence ensure that the point-of-care guidelines are followed and the processes are monitored for blood culture tests [2,6].

The checking of clinical information, such as the patient’s identity or doctor’s order, by using a mobile phone has been shown to improve workflow efficiency in clinicians [6]. However, to our knowledge, there is no mobile app that indicates the correct methods for blood sampling, monitors the process of sampling, and accordingly recommends quality improvement measures in blood culture tests. Recently, a mobile phone app for blood culture testing was developed at Asan Medical Center, a tertiary hospital in South Korea [30]. The “Blood Culture” app provides the information of patients who require blood culture tests and monitors the tests by recording the time of sampling, amount of blood sampled, and sampling sites. Before this, such data were not collected in the hospital. In this feasibility study, we implemented the app in medical intensive care units (MICUs) to assess the potential for its utilization in a clinical environment, by reviewing the usage data among a small group of users and by assessing their feedback and the data related to blood culture sampling.

## Methods

### Introduction to the Blood Culture App

The Blood Culture app was developed for iPod touch and iPhone (iOS version 5.1.1; Apple Inc) from June 2011 to June 2012 by a team of doctors from the departments of laboratory medicine, infectious diseases, emergency medicine, and biomedical informatics; a nurse; and 2 technicians from the medical information office. First, through an analysis of the process of blood culture test sampling, blood culture sampling parameters were defined to guide clinicians in the use of the Blood Culture app. The blood culture sampling parameters were defined based on 2 purposes: to monitor the process of blood culture test sampling (such as blood sampling sites, blood sampling volume, sampling time, and samplers’ names) and to support streamlined workflow at the point of care by checking the patient’s identity and doctor’s order in real time (such as the names of patients who needed blood culture tests, patient identification numbers, and blood culture test numbers). The processes of scanning the bar codes of the blood culture bottle and the patient identification band, as well as the process of entering the sampling parameters, were newly added to the blood culture sampling protocol.

**Figure 1:**
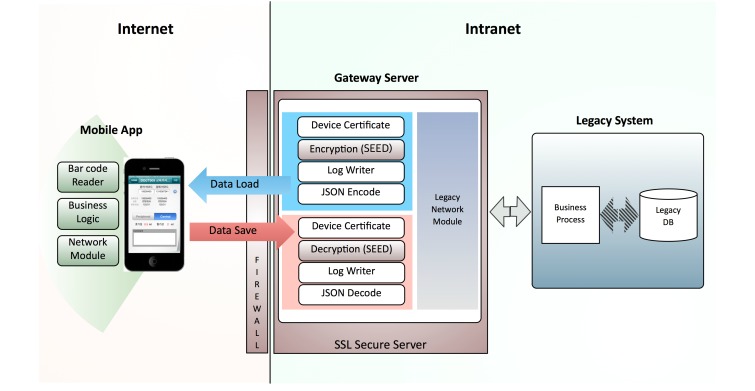
System architecture of the Blood Culture app service. The app can load patient and specimen data from the legacy system through a gateway server in the hospital, which enforces the security of the clinical data. The gateway server enables data exchange between the app and the legacy system. This gateway server prohibits direct access of the mobile client application to the legacy database via device certification and encryption functions. SEED is a 128-bit encryption algorithm. JSON: JavaScript Object Notation; SSL: Secure Sockets Layer.

With regard to features, the app enables matching between the prescribed blood culture test and the information of patients who need the test in real time, and it facilitates the entry of blood culture sampling parameters. Using a certified clinician’s identification number and password, participants could download the app from the research hospital’s app store via the research hospital intranet (Wi-Fi network). The app could be used on 3G (third-generation) and Wi-Fi networks. Using JavaScript Object Notation, the app communicates with the hospital gateway server, which prohibits direct access to the legacy database via device certification and encryption functions. Thereafter, the gateway server communicates with the legacy system (hospital information system; Figure 1).

**Figure 2:**
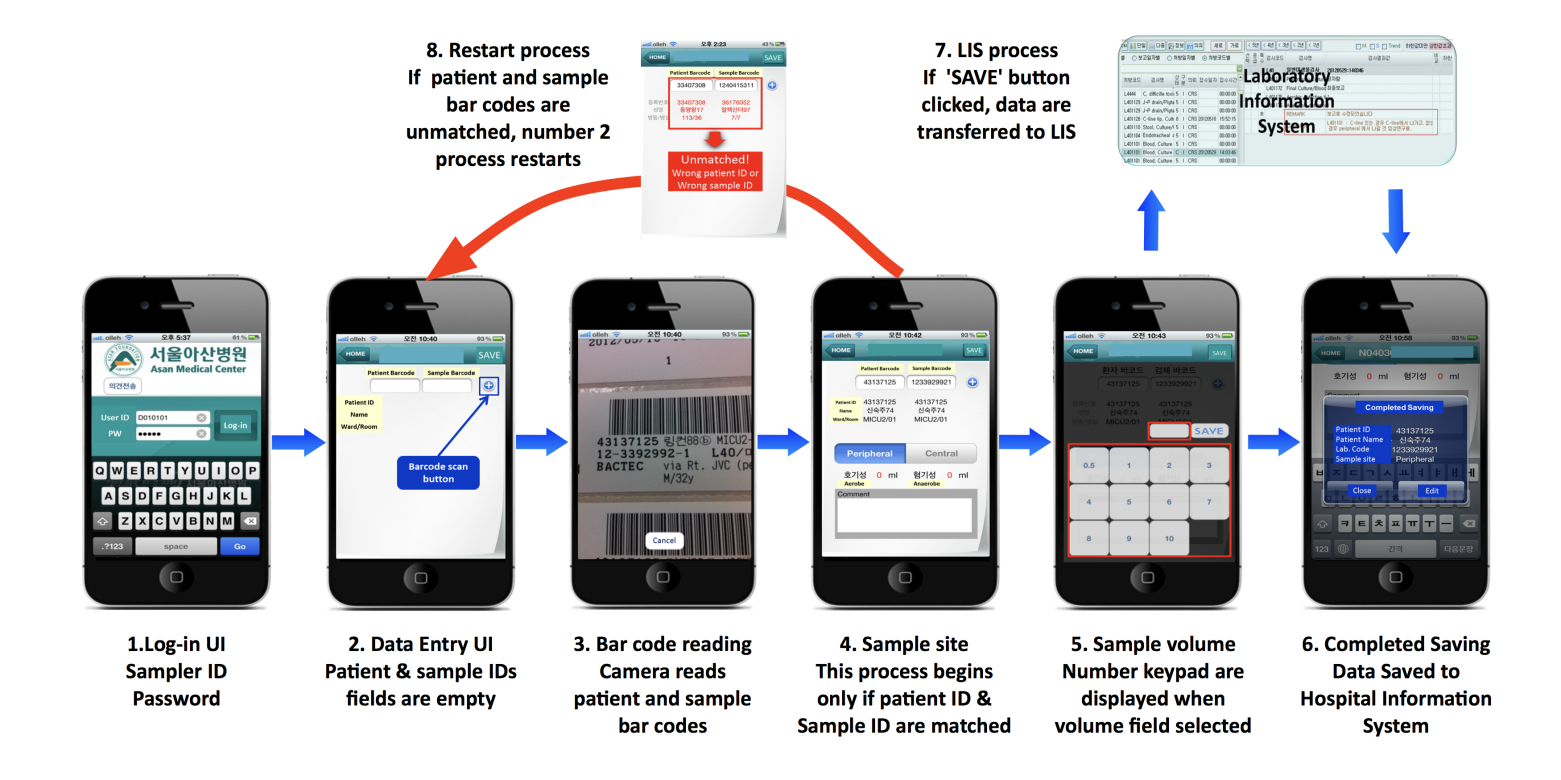
Service description of the Blood culture app. A sampler logs in to the Blood Culture app as a user (step 1). By using the mobile phone camera, the sampler scans the bar code on a patient’s wristband and blood culture test specimen, so the app can acquire the patient’s name and the patient identification (ID) number (steps 2 and 3). The app shows whether the bar codes match or not on the screen (steps 4 and 8). If not, the sampler is asked to rescan the bar codes (step 8). Once blood culture sampling is completed, the sampler enters and saves the blood culture sampling parameters into the app (step 5). The sampling parameters are stored in the hospital information system in real time (steps 6 and 7). UI: user interface; LIS: laboratory information system.

To ensure that the app functioned in a precise and quick manner in the clinical setting, the performance of bar code scanning with the iPod touch (fourth generation), iPhone 3GS, iPhone 4, and iPad 2 (Apple Inc) was tested by 3 doctors from the departments of laboratory medicine, infectious diseases, and emergency medicine, as well as by a nurse from the medical information office. The bar code scanning performance of the smart devices was found to be acceptable and no errors were noted during the performance test. To prevent sample contamination by a mobile phone, we educated users to match information between the prescribed blood culture test and the patients’ identification by scanning the bar code before blood sampling, proceeding with the blood sampling process using an aseptic technique, and then entering the blood culture sampling parameters. The protocol for using the Blood Culture app is illustrated in detail in Figure 2.

### Study Design and Setting

This study was conducted at our research hospital located in Seoul, South Korea, which has 2670 beds and a home-grown hospital information system (HIS). A computerized physician order entry method via a laboratory information system (LIS) was adopted in the early 1990s and electronic medical records were established in 2004 [30]. This feasibility study was conducted in 2 MICUs between July 4, 2012, and July 26, 2012, (over 3 weeks) by 4 medical interns with the iPhone 3GS. The 2 MICUs were selected by the app development team based on the frequent blood culture tests conducted and the critical nature of the results at those MICUs. The Wi-Fi protocol used was the IEEE (Institute of Electrical and Electronics Engineers) 802.11a. One of the participants (doctor A) was involved in the study for only 7 days (July 20, 2012, to July 26, 2012) owing to dispatch to other hospitals before enrollment. The study participants who agreed to voluntarily participate in this study were selected and provided informed consent. The study was approved by the institutional review board of the hospital.

### Data Analysis

We collected log data from the participants to determine the usage pattern, including compliance and data accuracy, as well as the subjective opinions of the participants to assess the expected effects of the app, such as patient safety, timeliness, and efficiency. The log data were collected and saved from the app and also included blood sampling sites, blood sample volume, sampling time, and samplers’ identification numbers. Compliance was determined based on the completeness of the blood culture sampling parameters, whereas data accuracy was determined based on the error reports from users regarding whether a mismatch occurred between the entered data and the data shown in the app. The subjective assessments of the participants were collected primarily via a written survey with an open-ended questionnaire on their satisfaction with and suggestions for the app; moreover, face-to-face or telephone interviews were conducted with the 4 participants individually within 10 minutes to test the accuracy of the survey. The user survey was administered to the doctors only after their MICU rotations to avoid any biased opinions and owing to concerns that the survey could influence their performance records.

The descriptive analyses of the app usage and the blood culture sampling parameters were conducted using SPSS version 18.0 statistical software package (IBM Corporation).

## Results

### Blood Culture App Data

**Figure 3:**
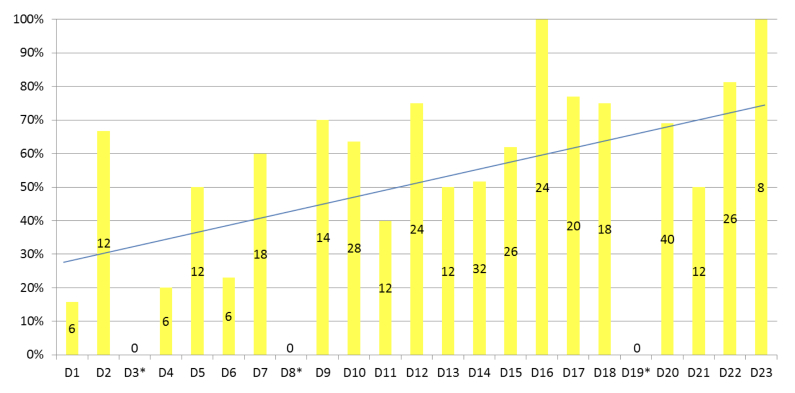
Daily usage frequency of the Blood culture app. All participants were on leave on D3, D8, and D19 (asterisk). The Blood Culture app was used for blood culture testing a total of 356 times (356/644 times, 55.3%) over 3 weeks—an average of 15.5 times/day. D represents the days during the study period.

**Figure 4:**
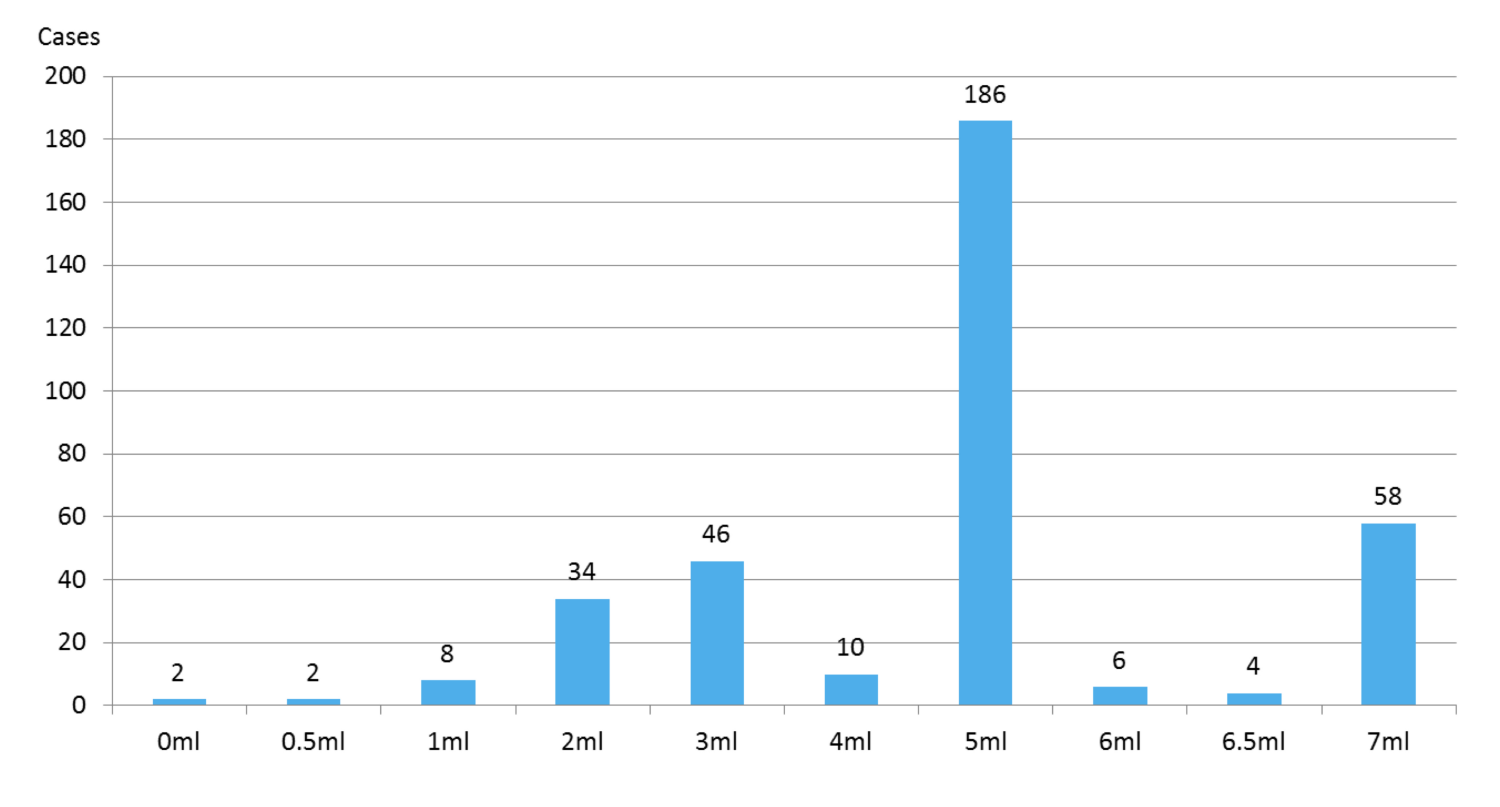
Distribution of blood culture sample volume data recorded by the Blood Culture app. A total of 5-7 mL of blood was collected in 254 cases (254/356 cases, 71.3%), and the mean volume was 4.6 (SD 1.6) mL.

The Blood Culture app was used to record the blood culture tests in clinical practice a total of 356/644 times (55.3% of all cases) over 3 weeks—an average of 15.5 times per day. A total of 644 blood culture tests were conducted in the MICUs during the study period. The daily use frequency of the app is shown in Figure 3, and the frequency of use gradually increased as the study progressed. The distribution of the entered blood culture sampling parameters is illustrated in Figure 4. In particular, 5-7 mL of blood was collected from 254/356 cases (71.3%), with a mean volume of 4.6 (SD 1.6) mL per bottle (Table 1), and samples were collected via the peripheral veins in 256/356 cases (71.9%). The sample volumes differed among the participants. Although blood sampling by doctor B was sufficient in all cases, blood sampling by doctor A was insufficient in all cases; however, the reason could not be ascertained.

To determine the compliance of entering the blood culture sampling parameters, the entry of all the parameters was carefully assessed. All the parameters were entered in 354/356 cases (99.4%) but not in 2/356 cases (0.6%) where the blood sample volume was recorded as 0 mL (the default value of the volume field). The users were asked if they entered the volume field correctly in order to assess whether there were any errors in the data saving stage for small values, and the users specified whether the data shown accurately reflected the data entered. No differences between the entered data and data shown in the app were reported by the users. In addition, no abnormal values were observed in the LIS.

**Table 1:** Comparison of blood culture sample volume and sampling site data recorded by 4 medical interns (N=356).

Parameters		Doctor A n (%)	Doctor B n (%)	Doctor C n (%)	Doctor D n (%)	Sum n (%)
**Blood culture sample volume per bottle (mL)^a^**						
	Mean (SD)	2.4 (0.6)	6.2 (1.4)	4.7 (0.7)	4.3 (1.6)	4.6 (1.6)
	<5	50 (100.0)	4 (4.3)	20 (21.7)	28 (23.0)	102 (28.7)
	≥5	0 (0.0)	88 (95.7)	72 (78.3)	94 (77.9)	254 (71.3)
**Blood culture sampling site**						
	Peripheral vein	34 (68.0)	68 (73.9)	68 (73.9)	86 (70.5)	256 (71.9)
	Central catheter	16 (32.0)	24 (26.1)	24 (26.1)	36 (29.5)	100 (28.1)

^a^The blood volume fields that were not filled were considered as 0 mL (default value).

### Survey Regarding the Blood Culture App

The participants’ opinions of the app, including patient safety, timeliness, and efficiency were assessed. First, with regard to patient safety, all the participants stated that the app had positive effects due to double checking via bar code scanning with the mobile phone camera in real time. Some of the comments made by the users were as follows: “It was great that bar code scanning could confirm that the patient who needed the blood culture test was correct, in addition to checking the patient’s name card or calling out patient’s name,” and “As the app ensured double checking of the patients and specimens, I was able to pay greater attention to the blood sampling.”

With regard to timeliness, differing opinions were noted among the participants (2 positive opinions and 2 negative opinions). However, the negative opinions were not related to the app itself but were instead related to the network environments in the hospital. Some of the comments made by the users were as follows: “The speed of bar code scanning of the patient wrist bands and specimens was fine,” and “The slow loading time and time for user login into the app due to the Wi-Fi connection were a hindrance.”

With regard to the efficiency, no positive comments were noted, possibly because a new process for entry of blood culture sampling parameters was added to the overall protocol. Of the participants, 2 reported that they were unsure whether the app enabled efficiency, whereas the other 2 participants reported negative opinions. Some of the comments made by the users were as follows: “If the work of entering the blood culture sampling parameters is made mandatory, then I would like to use the app. However, I am current not sure about the need for inputting the blood culture sampling parameters,” and “I have many things to do during the day. Do I also need to enter blood culture sampling parameters such as blood sample volumes and sites in addition to my daily tasks?”

The participants also provided suggestions for improvement of the app, including features such as screen layout and input mode, integration of the app with the HIS, and the hospital intranet. Some of the comments made by the users were as follows: “I would like to verify that the entered blood culture sampling parameters are stored correctly in the LIS,” and “I would like to view the blood culture results on the app as well as on the LIS.”

## Discussion

### Principal Findings

In this feasibility study, we found that the compliance to data entry was satisfactory (354/356, 99.4%) in the clinical setting. No error related to data entry via the app was noted. With regard to the satisfaction level and expectation of effectiveness, all the participants reported positive opinions. However, improvements in the network environment and work process policy were requested for improving timeliness and efficiency.

Although only a small group was tested, the Blood Culture app was found to promote patient safety by the users. Patient identification support and improvement of the blood culture test quality could further enhance patient safety. However, to improve test quality, it is important to educate and guide clinicians as the blood sampling performance could affect the accuracy of the test [18,21,31]. In particular, the volume of sampled blood is the most important factor influencing a correct result [13,15,26]. Mermel and Maki [26] reported that insufficient volume collection often occurs because only a few clinicians and nurses are aware of the vital influence of collection volume on blood culture sensitivity. Hence, increasing the awareness of clinicians regarding this aspect during the point-of-care process and management of test quality represent important solutions. The Blood Culture app was developed for such purposes at the point of sampling. In our study, insufficient collection was noted in 28.7% (102/356) of the cases, although most cases (254/356, 71.3%) showed sufficient blood volume collection (5-7 mL). Accordingly, information on blood volume could be used as an index of reliability. The Blood Culture app can also be used to provide appropriate feedback and to reeducate samplers with relatively frequent errors. In fact, the participants also requested feedback regarding their blood culture quality during the survey.

### Comparison With Prior Work

The Blood Culture app described herein differs from other existing medical apps. It directs the actions of clinicians, helps clinicians identify patient information and enter patient-related data in an app connected to the HIS, and monitors the activity of clinicians for quality improvement. Thus, the app can be used to improve patient safety, timeliness, and efficiency for blood culture testing. To guide clinicians more effectively, the app can be upgraded to provide information on the steps for disinfecting hands and disinfecting skin, as well as knowledge about the sterile glove technique. The effective implementation of the app can reduce the gap between the guidelines and actual clinical practice. Consequently, the quality control of the blood culture process could improve patient outcomes, reduce inappropriate antibiotic use and antibiotic resistance, and promote treatment efficiency.

The times for blood culture order, sample submission, and reporting of results have been routinely recorded at our research hospital. However, blood culture sampling parameters—essential data for blood culture quality control—are not collected and managed. The Blood Culture app attempted to collect such information at the point of blood sampling. The speed of the app and ease of data entry were considered to be good, although 2 limitations were cited—weak wireless network environment and the need for data entry. Slow loading time and log-in delay occurred because of the weak wireless network environment or communication with the HIS. These can be overcome by improving the network environment of the hospital and adding an automatic log-in or touch ID feature with the app.

However, the need for recording the blood culture sampling parameters cannot be emphasized without a change in the internal hospital policy regarding the collection of such information to improve test quality. Without such a policy, the app could be considered inefficient and unnecessary. In the departments of laboratory medicine and infectious diseases in the hospital, the policy regarding the recording of blood culture sampling parameters was obligatorily revised, although the change was only recently finalized. Once it is established and appropriately introduced, the app could be widely used to record information correctly and promptly. However, it may be more efficient to record such information via a desktop computer, depending on the sampler’s memory after the procedure. In fact, a desktop version and upgrade versions (for Apple’s iOS and Google’s Android operating systems) of the app were developed and implemented for computerized physician order entry in April 2013.

### Lessons Learned

We determined the features that could ensure active use of the app in clinical practice: app functionality for users, high-speed and seamless wireless network, and favorable internal policy. The app can be upgraded to provide more information regarding appropriate blood culture techniques and feedback on the user’s test quality, which could improve the skills of the samplers. A high-speed wireless network and seamless connection to the HIS are essential for its use in the point-of-care settings; the lack of such utilities could cause frustration for users. In addition, an internal policy regarding the recording of blood culture sampling parameters should be established to manage and improve blood culture quality. Strategies to manage such data and guide clinicians could consequently improve the quality of the tests. Our findings may also be useful for individuals developing and implementing mHealth apps in the clinical environment.

### Limitations and Future Studies

This study had certain limitations, including the small number of participants, short study period, and single study site. Although the study findings indicated the potential for mobile app implementation in point-of-care settings, the effects of the app on sample volume, patient identification, or contamination rate were not assessed. To control the contamination rate, the app should include aseptic technique guidelines or a program for auditing the data on contaminated blood culture samples; however, it would be conducted in a manner that does not involve apportioning blame. With regard to blood sampling, there is a possibility of overrecording by samplers; however, the participants did not receive any penalty for insufficient sample volume in this study. If an internal policy recommends a penalty for such cases, the samplers may tend to overrecord the sample volume. In those cases, the app cannot be used for quality control. Hence, another solution, such as automatic blood culture volume measurement in the laboratory, is needed. Moreover, we could apply the app’s features, including checking the patient’s identity and doctor’s order in real time, to the sampling processes of other blood tests as well as blood transfusions and the administration of medications.

The data collected from the app, such as sample volume, sampling time, and sampling site of blood culture, could indicate quality improvements in the test, such as the measurement of guideline adherence and evidence of the hospital policy regarding sampling. Further studies that compare the conventional process with the new process (with the app) in terms of impact of contamination, blood volume, or patient identification would be useful for individuals managing hospital infection and implementing mHealth apps in clinical practice.

### Conclusions

The Blood Culture app can be applied in the clinic and can be used to provide real-time information, input patient data at the bedside, and manage blood sample quality. If internal policy makes the recording of blood culture sampling parameters an obligation, then clinicians would be more inclined to use the app than a desktop-based program.

## Abbreviations

HIS: hospital information system
IEEE: Institute of Electrical and Electronics Engineers
LIS: laboratory information system
mHealth: mobile health
MICU: medical intensive care unit
